# Europe’s War against COVID-19: A Map of Countries’ Disease Vulnerability Using Mortality Indicators

**DOI:** 10.3390/ijerph17186565

**Published:** 2020-09-09

**Authors:** Alexandra Horobet, Anca Angela Simionescu, Dan Gabriel Dumitrescu, Lucian Belascu

**Affiliations:** 1 Department of International Business and Economics, The Bucharest University of Economic Studies, 010374 Bucharest, Romania; alexandra.horobet@rei.ase.ro (A.H.); dan.dumitrescu@rei.ase.ro (D.G.D.); 2 Carol Davila University of Medicine and Pharmacy, Department of Obstetrics and Gynecology, Filantropia Hospital, 020021 Bucharest, Romania; 3 Department of Management, Marketing and Business Administration, Lucian Blaga University of Sibiu, 550024 Sibiu, Romania; lucian.belascu@ulbsibiu.ro

**Keywords:** COVID-19, comorbidities, mortality, Europe, eastern countries, western countries

## Abstract

Specific and older age-associated comorbidities increase mortality risk in severe forms of coronavirus disease (COVID-19). We matched COVID-19 comorbidities with causes of death in 28 EU countries for the total population and for the population above 65 years and applied a machine-learning-based tree clustering algorithm on shares of death for COVID-19 comorbidities and for influenza and on their growth rates between 2011 and 2016. We distributed EU countries in clusters and drew a map of the EU populations’ vulnerabilities to COVID-19 comorbidities and to influenza. Noncommunicable diseases had impressive shares of death in the EU but with substantial differences between eastern and western countries. The tree clustering algorithm accurately indicated the presence of western and eastern country clusters, with significantly different patterns of disease shares of death and growth rates. Western populations displayed higher vulnerability to malignancy, blood-related diseases, and diabetes mellitus and lower respiratory diseases, while eastern countries’ populations suffered more from ischaemic heart, cerebrovascular, and circulatory diseases. Dissimilarities between EU countries were also present when influenza was considered. The heat maps of EU populations’ vulnerability to diseases based on mortality indicators constitute the basis for more targeted health policy strategies in a collaborative effort at the EU level.

## 1. Introduction

Coronavirus disease (COVID-19) impact has brutally revealed vulnerabilities in countries’ responses, evident in the disease’s spread and virulence, and in terms of health policy measures. As of July 10, 2020, Europe held the second-highest number of deaths caused by the disease (196,475, 35.09% of all deaths) and the third-highest number of cases (2,558,747, 20.51% of the total, after America and Asia) [1]. With 3423.7 cases, 262.6 deaths per million inhabitants, and a case fatality ratio (CFR) of 7.67%, Europe is facing a critical situation. In the European Union, there were 179,192 COVID-19 deaths—a mean of 200.21 deaths per million inhabitants (*p* < 0.05), varying between 5.00 (Slovakia) to 844.00 (Belgium). Five countries (Belgium, United Kingdom, Spain, Italy, and Sweden) had more than 500 deaths per million inhabitants, and another three countries had more than 200 deaths (France, Netherlands, and Ireland). A stark contrast between the EU’s western and eastern parts was calculated; eastern countries had a mean of 41.36 deaths per million inhabitants (*p* < 0.05), while their western counterparts had a mean of 303 deaths (*p* < 0.05)—based on [1].

Mathematical models have demonstrated the swift, exponential spread of Severe Acute Respiratory Syndrome Coronavirus 2 (SARS-CoV-2) and have shown that the viral latency period and disease contagion are positively correlated [2]. Clinical features are very different, from asymptomatic to symptomatic cases. After an incubation period of 5 days, it is estimated that 81% of infected persons develop symptoms [2,3,4]. Death occurs in the symptomatic cases, and the time from symptom onset to death follows a log-normal distribution, with a mean of 20.2 days [5]. For those over 70 years and who have associated comorbidities, a rapid potential for progression to death from the onset of fever has also been estimated [6].

Financial efforts to fight the disease are remarkable: the World Health Organization Global Fund initiative (USD$1 billion funding) [7] or the EU research funds of €352.5 million [8], including vaccine, treatment, and test development. Customary epidemic community measures were also taken, including social distancing, wearing a mask, washing hands, quarantine, self-isolating, and partial or total lockdown [9], with disastrous economic and social consequences, and financial losses.

Using the model of influenza pandemics, clinical data reporting on severe forms of COVID-19 have highlighted associated cardiovascular diseases, neoplasia, diabetes, obesity-related comorbidities, and an age above 65 years as significant mortality risk factors [10,11,12,13]. The concept of “vulnerability” has been introduced since the early 1990s to study the impact of natural disasters on populations, as a test of society’s ability to make vulnerable populations resilient when disaster strikes [14,15]. In order to address populations’ vulnerabilities to diseases, the WHO has developed tools and guidance to support countries’ efforts against the expansion of noncommunicable diseases [16]. EU health policies have focused so far on reducing known risk factors associated with chronic diseases, smoking, obesity, and binge drinking as significant determinants of the leading causes of death: cardiovascular diseases and cancer. At the same time, the EU has implemented guidelines and have encouraged the finding of new treatment molecules. 

Still, new causes of death and risk factors were reported, yet there are unexpected connections between communicable (CDs) and noncommunicable diseases (NCDs) [17,18]. NCDs hold a higher toll on the number of deaths than CDs; 71% of all deaths globally occur from NCD diseases, of which 15 million people are between 30 and 69 years [19]. NCDs have overtaken CDs and are the leading causes of global mortality and morbidity; according to WHO data, 71% of all deaths at the global level were caused by NCDs in 2018 [20]. While significant advances against CDs have been made, there is insufficient progress in preventing and controlling NCDs [21]. The time spent when placed in special measures for the vulnerable population may be associated with the proportion of intensive care units (ICU) admissions and deaths associated with the COVID-19 pandemic [22].

Our study’s objectives reside in mapping EU countries based on their populations’ vulnerability to COVID-19 comorbidities and in further associating these vulnerabilities with COVID-19 and influenza incidence. Influenza’s importance as a cause of death might provide valuable insight into countries’ preparedness before the COVID-19 outbreak. To our present knowledge, this is the first systematic and statistically enforced approach towards grasping EU countries’ vulnerability to COVID-19 comorbidities as a useful tool for appropriate management strategies to be implemented.

## 2. Methods

Causes of death for the 28 EU member countries (as of December 31, 2019) from the European Mortality Database and Eurostat between 2011 and 2016 were matched with 13 comorbidities for hospitalized patients for COVID-19 disease [23,24]. The total population we cover in this study is 507,070,197, of which 5,590,916 are deaths (as 2011–2016 averages). We have selected 13 comorbidities described in Tomlins et al. (2020) found in the case of 95 sequential hospitalized patients in the United Kingdom diagnosed with COVID-19 [12]. Further, we matched them to the best with the causes of death for which data was available, using the International Statistical Classification of Diseases and Related Health Problems 10th Revision (ICD-10) [25]; see Table 1. Moreover, we have collected data on influenza-caused deaths (influenza (including swine flu) J09-J11 ICD-10 code) for 27 out of 28 EU countries—Malta was excluded due to lack of data availability (i.e., the Malta has not provided data on deaths caused by influenza between 2011 and 2016). Data on causes of death was collected for the total population and the population aged 65 years for the 2011–2016 period, for all genders. 

We calculated the mean share of death (SOD)—the percentage of deaths attributed to a specific cause of death in the total number of deaths—and its compound annual growth rate (CAGR) between 2011 and 2016. The CAGR was calculated as follows:

$$\mathrm{CAGR}=\left[{\frac{{\mathrm{Share}\;\mathrm{of}\;\mathrm{death}}_{2016}}{{\mathrm{Share}\;\mathrm{of}\;\mathrm{death}}_{2011}}}^{\left(1/5\right)}\right]-1$$

We further used them to apply a tree clustering algorithm to identify similarities and differences between EU countries based on two data sets, without knowing a priori the belonging of countries to these clusters:

(1) thirteen COVID-19 comorbidity SODs and their corresponding annual growth rates, for the total population and the population aged 65, and
(2) influenza SODs and their corresponding annual growth rates, for the total population and the population aged 65.

The tree classification implemented in a C&RT (Classification and Regression Tree) framework [26] iteratively builds the clusters and provides a heat map of the resulting clusters based on the values of the variables included in the analysis, i.e., SODs and their corresponding CAGRs. Thus, we found the distribution of EU countries in clusters by taking into account the importance of COVID-19 comorbidities as SOD, which further allows us to draw the map of EU in terms of populations’ vulnerabilities to COVID-19 comorbidities. Moreover, we applied the same clustering algorithm to influenza SOD and CAGR and further contrasted the results with COVID-19 impact across EU countries. The algorithm uses Euclidean distances and Ward’s amalgamation method for identifying clusters. These are two frequently used parameters of statistical clustering algorithms. The Euclidean distance was calculated as the geometric distance between variables x and y in a multidimensional space:

$$\mathrm{Distance}\left(x,y\right)={\left\{\sum_{i}^{}{\left(x_{i}-y_{i}\right)}^{2}\right\}}^{1/2}$$

where i designates the case for which the distance is calculated. Ward’s method of amalgamation uses an analysis of variance (ANOVA) approach to assess the distance between the clusters; specifically, the method minimizes the sum of squares (SS) of any clusters that can be potentially formed at each step of the algorithm [27,28]. All data have been standardized before applying the tree clustering algorithm. The optimal number of clusters for each data set was determined by observing the dendrogram resulting from iterative clustering and by using the Pseudo F Index [29]. The Pseudo F statistic is the ratio of between-cluster variance to within cluster variance:

$$\mathrm{Pseudo}\;F=\frac{\mathrm{GSS}/\left(k-1\right)}{\mathrm{WSS}/\left(n-k\right)}$$

where n is the number of observations, k is the number of clusters formed at any step in the hierarchical clustering, GSS designates the between-group sum of squares, and WSS represents the within group sum of squares. This is one of the best techniques for determining the optimal number of clusters in a dataset and is widely used in healthcare research [30].

## 3. Results

**Figure 1:**
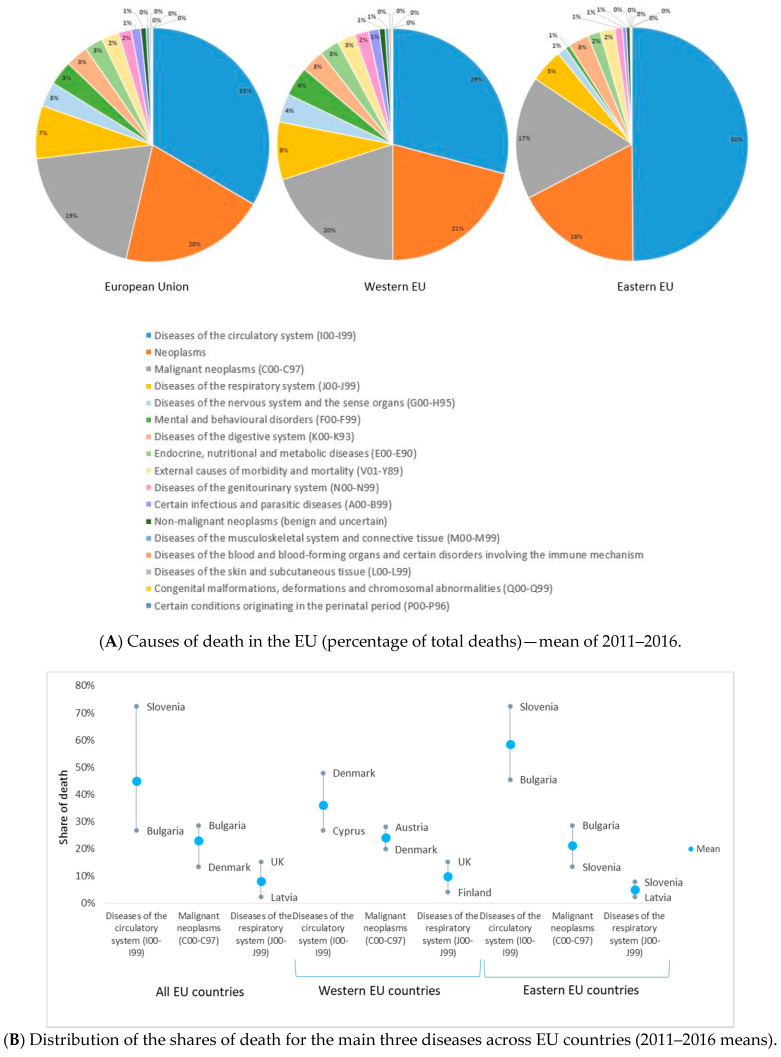
Causes of death in the European Union, 2011–2016. (1) Noncommunicable diseases held high shares of death in the EU between 2011–2016, mostly for diseases of the circulatory system (39%), malignancy (27%), and diseases of the respiratory system (8%). Eastern countries recorded 53% share of death (SOD) for circulatory system diseases compared to their Western counterparts (36%) but lower SOD for malignant neoplasms (24% against 29%). (2) Causes of death for all European Union countries were collected from the Eurostat and European Mortality Database, according to the codes of the International Classification of Diseases, 10th Revision (ICD-10). They cover all ages and genders, on a population of 507,070,197 persons (mean of 2011–2016). The mean annual number of deaths in the European Union for all causes (except injury, poisoning, and certain other consequences of external causes (S00–T98)) is 5,590,916 people. There were 28 European member countries at the end of 2016, divided into two groups: the Western group (17 countries: Austria, Belgium, Cyprus, Denmark, Finland, France, Germany, Greece, Ireland, Italy, Luxembourg, Malta, Netherlands, Portugal, Spain, Sweden, and United Kingdom), with an average population between 2011 and 2016 of 403,478,671 persons, and the Eastern group (11 countries: Bulgaria, Croatia, Czechia, Estonia, Hungary, Latvia, Lithuania, Poland, Romania, Slovakia, and Slovenia), with an average population between 2011 and 2016 of 103,591,526 persons. Panel **A** presents the distribution of causes of death among all EU countries, in Western EU countries, and in Eastern EU countries (from left to right). Panel **B** shows the distribution of the leading three causes of deaths as a percentage of the total number of deaths (all age groups and all genders) across the 28 EU countries. (3) Share of death represents the percentage of deaths attributed to a specific cause of death in the total number of deaths (measured in %).

In the EU, NCDs had impressive shares of death between 2011–2016, particularly for diseases of the circulatory system (39%), malignancy (27%), and diseases of the respiratory system (8%), but with rather wide variations between countries (Figure 1A,B). Eastern countries recorded 53% SOD for circulatory system diseases compared to their Western counterparts (36%) but lower SOD for malignant neoplasms (24% against 29%). Some countries recorded soaring SOD values for the first two most important comorbidities: circulatory system diseases in Bulgaria (66.24%) and Romania (59.51%), and malignancy in Slovenia (31.41%) and the Netherlands (30.86%). 

### 3.1. COVID-19 Comorbidities—Data Description

Of the 13 studied comorbidities, the SODs were above 5% for malignancy, ischemic heart diseases, other heart diseases, cerebrovascular diseases, and other circulatory diseases, for both population categories. Malignancy had the highest SOD across EU populations, with means of 25.39% (*p* < 0.05) for the total population and 22.98% (*p* < 0.05) for the population over 65, followed by ischemic heart diseases (means of 15.86% (*p* > 0.05) for the total population and 17.31% (*p* > 0.05) for population above 65 years) (Table 2). The *t*-test statistic applied to dependent samples for each disease in the total population and the population over 65 has shown that SOD means are statistically significantly different between the total population and the population aged over 65 years for 10 out of the 13 COVID-19 comorbidities as well as for influenza. The exceptions were blood diseases, nervous system diseases, and asthma.

Comorbidities with low SOD were asthma (0.13% (*p* < 0.05) and 0.14% (*p* > 0.05)) and other endocrine diseases (0.55% (*p* > 0.05) and 0.51% (*p* > 0.05)). Generally, SODs for COVID-19 comorbidities were higher as mean and median in the population aged 65. Cerebrovascular diseases, other circulatory diseases, and other lower respiratory diseases have shown higher SODs for the population aged 65 for all 28 EU countries, and 27/28 countries had higher SODs for blood diseases and genitourinary diseases in the population aged 65. All countries had higher malignancy SODs for the total population compared to the population aged 65. 

**Figure 2:**
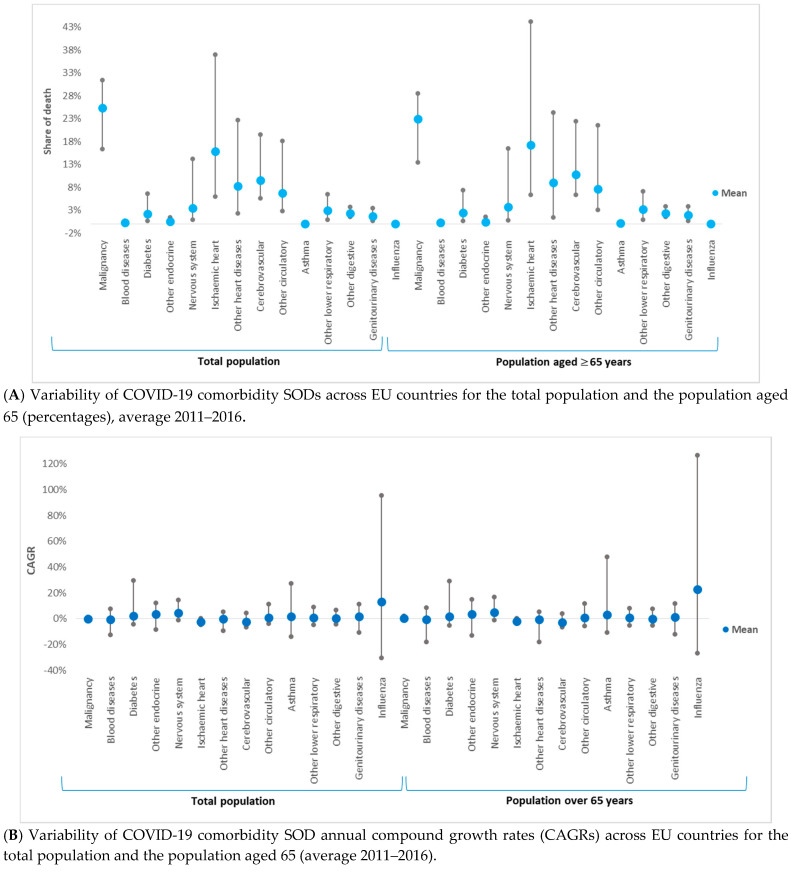
Variability of 13 COVID-19 comorbidities and influenza shares of death (SODs) and compound annual growth rates between 2011 and 2016 (CAGR) across 28 European Union countries and for two population categories: the total population and the population aged above 65 years. (**A**) (1) Share of death represents the percentage of deaths attributed to a specific cause of death in the total number of deaths (measured in %). (2) The *t*-test statistic applied to dependent samples has shown that SOD means are statistically significantly different between total population and population aged ≥ 65 years for 10 out of the 13 coronavirus disease (COVID-19) comorbidities and for influenza (*p* < 0.05). The exceptions are blood diseases, nervous system diseases, and asthma (*p* > 0.05). (**B**) (1) SOD variabilities for ischemic heart diseases, other heart diseases, and other circulatory diseases were the highest across EU countries, while asthma and blood diseases had the lowest variability. The growth rate of comorbidities’ SOD (CAGR) varied between countries and comorbidities; only three diseases have seen, on average, a decline in SOD for both population categories: cerebrovascular diseases, ischemic heart diseases, and blood diseases. (2) Compound annual growth rate (CAGR) represents the compound annual growth rate in shares of death between 2011 and 2011. (3) The *t*-test statistic applied to dependent samples has shown that CAGR means are statistically significantly different between the total population and the population aged ≥ 65 years for 8 out of the 13 COVID-19 comorbidities and for influenza (*p* < 0.05). The exceptions are other endocrine diseases, other heart diseases, asthma, other digestive diseases, and genitourinary diseases (*p* > 0.05).

SOD variance ranking across countries was similar between the two categories of population (Figure 2A). SOD variabilities for ischemic heart diseases, other heart diseases, and other circulatory diseases were the highest across EU countries (Table 2 and Figure 2A). Asthma and blood diseases had the lowest variability.

The growth rate of comorbidities’ SODs (CAGRs) varied between countries and comorbidities (Table 3 and Figure 2B). Three diseases have shown, on average, a decline in SOD for both population categories: cerebrovascular diseases (−2.406% (*p* > 0.05) and −2.890% (*p* > 0.05)), ischemic heart diseases (−2.324% (*p* > 0.05) and −2.044% (*p* > 0.05)), and blood diseases (−0.356% (*p* > 0.05) and −0.615% (*p* > 0.05)). The remaining ten comorbidities had, on average, increasing SODs; the highest was for other endocrine diseases (3.489% (*p* > 0.05) and 3.689% (*p* > 0.05)). For asthma, nervous system diseases, malignancy, ischemic heart diseases, and other endocrine diseases, SOD mean and median growth rates in the total population were higher than the growth rate for the population over 65 years. The *t*-test statistic applied to dependent samples has shown that CAGR means were statistically significantly different between the total population and the population aged over 65 years for 8 out of the 13 COVID-19 comorbidities and for influenza. The exceptions were other endocrine diseases, other heart diseases, asthma, other digestive diseases, and genitourinary diseases.

Asthma had the highest SOD growth rate in both population sets in Greece (27.24% and 46.75%), and diabetes had the highest in Malta (29.84% and 29.29%). Greece also had a very high CAGR in both population categories for nervous system disease SOD (14.78% and 17.09%) and other circulatory diseases (11.22% and 11.71%). Czechia recorded the highest CAGR for other endocrine diseases SOD for the total population, and Poland had the highest for the population aged 65. Several countries have shown substantial progress in managing these diseases, as their SOD between 2011 and 2016 has declined. Blood diseases’ SOD had the highest negative CAGR for Slovakia (−12.24% for the total population and −17.87% for the population above 65 years), and asthma SOD had the highest negative growth rate for Luxembourg (−13.64% and −10.8%). Overall, eastern EU countries have recorded higher mean CAGRs for 10/13 SODs in the total population and 11/13 SODs in the population above 65 years.

### 3.2. Influenza—Data Description

The mean SODs for influenza across EU countries were low, 0.0725% (*p* < 0.05) for the general population and 0.065% (*p* < 0.05) for the population aged 65 years, but the medians were smaller for both populations. Sweden recorded the highest influenza SOD (0.18% and 0.17%), and Romania had the lowest (0.009% and 0.004).

The mean influenza SOD growth rates in the EU were 13.13% (*p* < 0.05) for the total population and 22.73% (*p* < 0.05) for the population aged 65 years; 20/27 countries recorded positive growth rates of influenza SOD for the total population, and 21/27 countries recorded positive growth rates for the population aged 65. Slovenia had the highest growth rate for both populations: 95.69% and 126.52%, respectively. Seven countries have decreased influenza SOD for their total population, and six countries had decreases for the population aged 65. Cyprus is an interesting case, as influenza SOD for the total population declined (−13.57% CAGR) but that for the population aged 65 increased (7.31% CAGR).

Although influenza SOD was generally higher for the total population compared to the population aged 65 in most EU countries (except Belgium, France, Luxembourg, and Slovenia), the growth rate of influenza SOD for the population aged 65 was higher for 23/27 EU countries (except for Bulgaria, Czechia, Latvia, and Luxembourg).

### 3.3. Results of Tree Clustering Algorithms

#### 3.3.1. Clusters Based on COVID-19 Comorbidity SOD and CAGR

**Figure 3:**
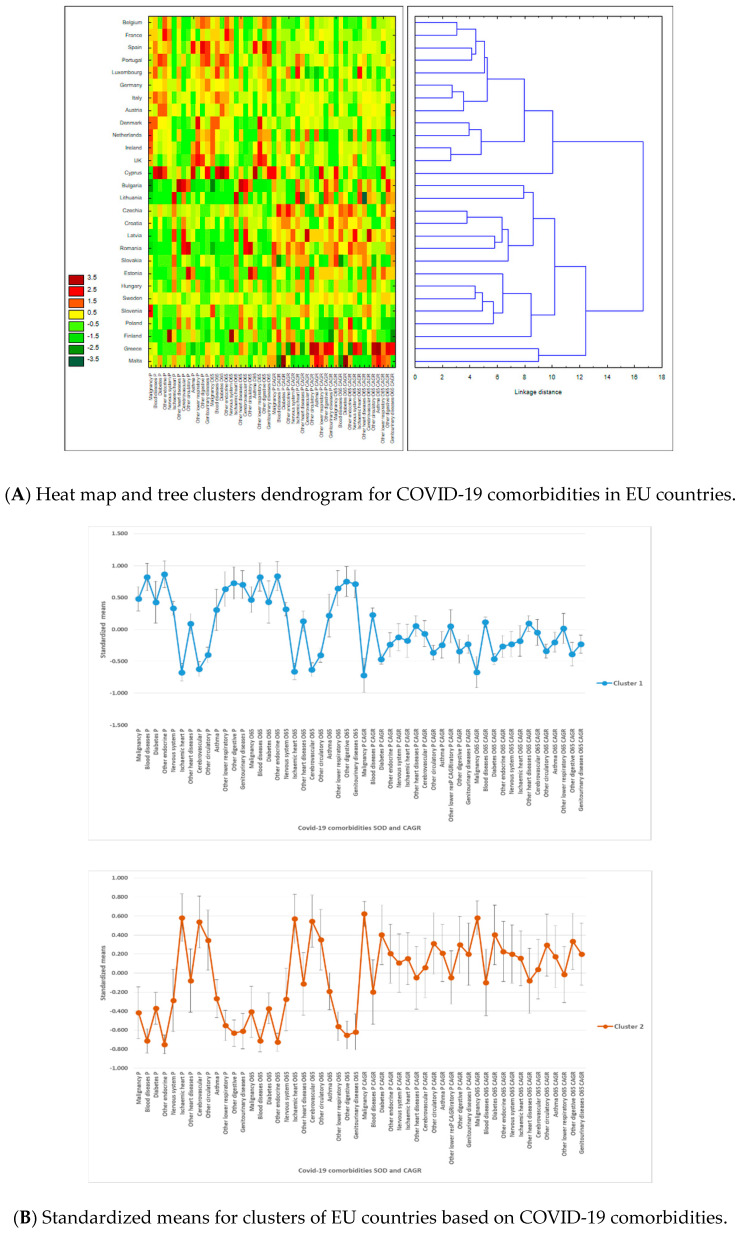

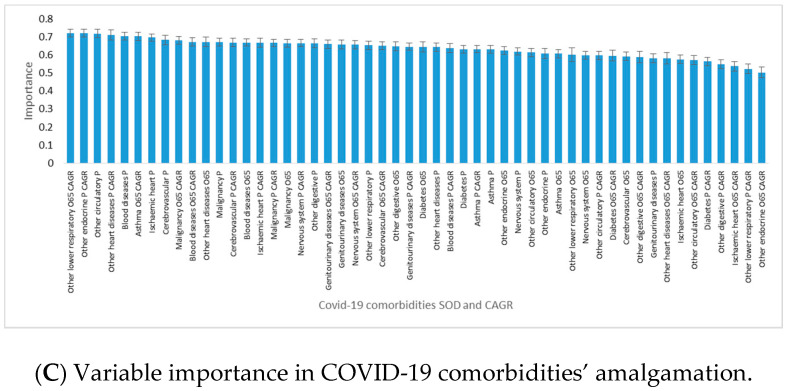
Results of tree clustering of COVID-19 comorbidities for 28 European Union countries: heat maps, cluster attributes, and variable importance. Tree clustering results are based on Euclidian distances and the Ward’s amalgamation method applied on shares of death (SOD) and compound annual growth rates (CAGR) for all 28 EU countries, on mean values between 2011 and 2016. Panel **A** depicts on the left the heat map of EU countries in terms of SOD and CAGR—greener cells indicate lower standardized values, and darker red cells indicate higher standardized values—for each of the 13 COVID-19 comorbidities. Areas of the heat map coloured in green indicate lower vulnerability, and areas coloured in red indicate higher vulnerability to diseases, measured in SOD and/or CAGR. The right side of the panel shows the dendrogram of countries’ distributions in clusters based on the linkage distance (the distance between clusters’ members). Panel **B** presents the standardized (or normalized means, i.e., means on adjusted values to scale) of clustering variables (SOD and CAGR for the 13 COVID-19 comorbidities) for the two statistically significant clusters and the error bars based on standard error. Cluster 1 has 13 members, and cluster 2 has 15 members. Panel **C** presents the variables’ importance for predicting cluster membership via the C&RT/boosted tree algorithm, based on 100 repeatedly drawn samples via bootstrapping. The closer to 1 the variable is, the higher its importance for countries’ inclusion in one of the two clusters. Error bars in Panel **C** are based on standard deviations of variable importance. There are two clusters of EU countries based on their populations’ vulnerability to COVID-19 comorbidities: cluster 1 includes 13 western EU countries, and cluster 2 includes 11 eastern countries and 4 western countries (Sweden, Malta, Finland, and Greece). Countries in cluster 1 have higher SOD of all diseases than cluster 2, except for ischemic heart, cerebrovascular diseases, and other circulatory diseases, but lower growth rates of diseases SOD, except for blood diseases, other heart diseases, and other lower respiratory diseases, for both population sets. Overall, SODs were more important than their CAGR for countries’ assignments to clusters, but the highest power of differentiation between western and eastern EU countries belongs to diseases with high CAGRs among the elderly population.

Our tree clustering algorithm indicated the presence of two clusters (Figure 3A). The first cluster included 13 western EU countries, and the second cluster was formed of 11 eastern countries and 4 western countries (Sweden, Malta, Finland, and Greece). These two clusters were clearly differenced by their attributes (Figure 3B). The first cluster showed higher SODs for all diseases, compared to the second cluster, except for ischemic heart, cerebrovascular diseases, and other circulatory diseases. Countries in the first cluster had lower growth rates of disease SOD, except for blood diseases, other heart diseases, and other lower respiratory diseases, for both population sets.

In the first half of the amalgamation schedule, the majority of country pairs were between western countries (Ireland–United Kingdom, Germany–Italy, and Belgium–France), suggesting stronger similarities between them compared to eastern countries. This result has confirmed that disease SOD patterns were significantly different in the two parts of the EU.

Overall, SODs were more important than their CAGRs for country assignments to clusters (Figure 3C). The highest power of differentiation between western and eastern EU countries came from diseases with high CAGRs among the elderly population: other lower respiratory diseases, asthma, malignancy, and blood diseases. They were accompanied by other endocrine diseases’ CAGR in the total population and SOD in the total population for other circulatory diseases, ischemic heart, blood diseases, and other circulatory diseases.

#### 3.3.2. Clusters of Influenza SOD and CAGR

**Figure 4:**
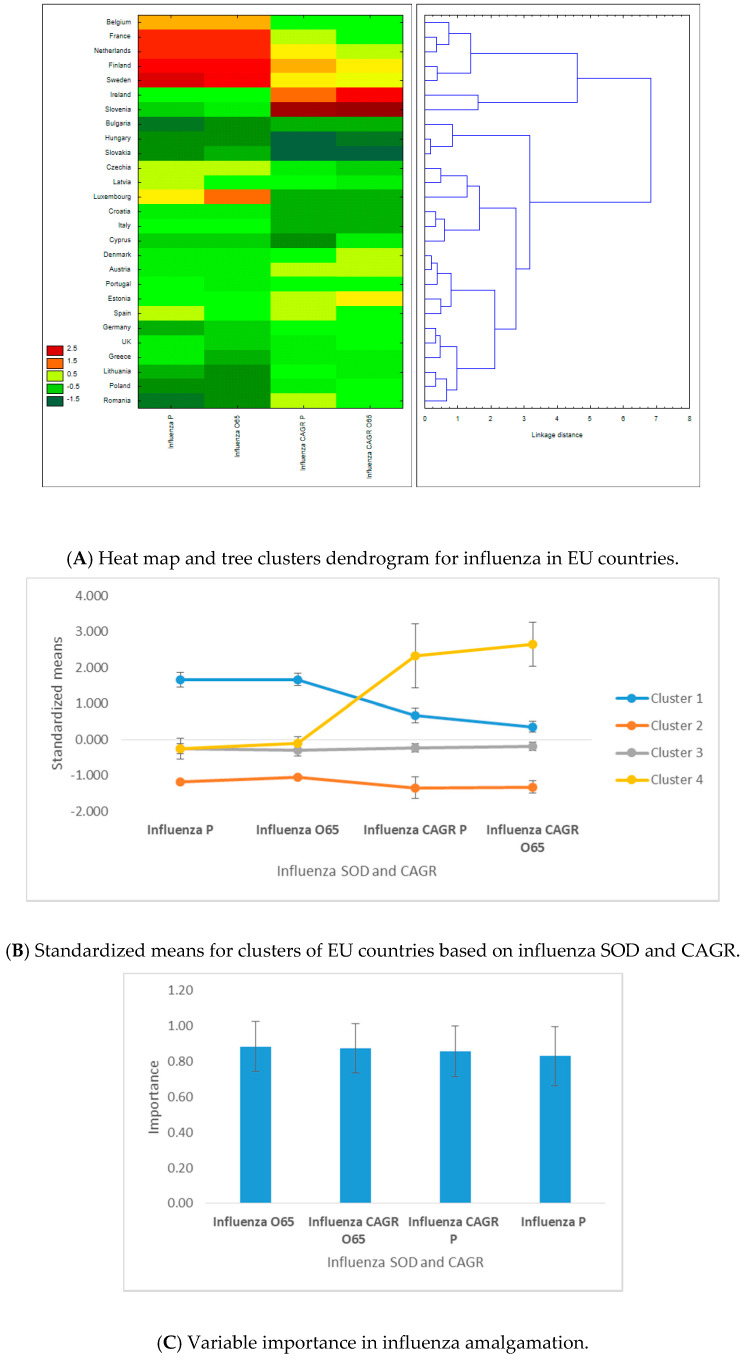
Results of C&RT tree clustering of influenza for 28 European Union countries: heat maps, cluster attributes, and variable importance. (1) Tree clustering results are based on Euclidian distances and the Ward’s amalgamation method applied on shares of death (SOD) and compound annual growth rates (CAGR) for 27 EU countries (Malta was excluded due to lack of data availability) on mean values between 2011 and 2016. Panel **A** depicts on the left the heat map of EU countries in terms of SOD and CAGR—greener cells indicate lower standardized values, and darker red cells indicate higher standardized values—for influenza. Areas of the heat map coloured in green indicate lower vulnerability, and areas coloured in red indicate higher vulnerability to diseases, measured in SOD and/or CAGR. The right side of the panel shows the dendrogram of countries’ distribution in clusters based on the linkage distance (the distance between cluster members). Panel **B** presents the standardized (or normalized means, i.e., means on adjusted values to scale) of clustering variables (SOD and CAGR for influenza) for the two statistically significant clusters and the error bars based on standard errors. Cluster 1 includes 5 members, cluster 2 includes 3 members, cluster 3 includes 17 members, and cluster 4 has 2 members. Panel **C** presents the variables’ importance for predicting cluster membership via the C&RT/boosted tree algorithm, based on 100 repeatedly drawn samples via bootstrapping. The closer to 1 the variable is, the higher its importance for countries’ inclusion in one of the two clusters. Error bars in Panel **C** are based on standard deviations of variable importance. (2) There are four clusters based upon influenza SOD and CAGR. Cluster 1 includes 5 western countries, with the highest influenza SOD and second-highest CAGR of influenza SOD, for both population sets. Cluster 2 includes 3 eastern countries, with the lowest influenza SOD and CAGR for both population categories. Cluster 3 includes 17 countries, except for Slovenia and Ireland, with low influenza SOD but very high SOD growth rate, which belong to cluster 4. Of the four clustering variables, influenza SOD and CAGR for the population above 65 years have slightly higher predictive power for countries’ assignments into clusters.

There were four clusters based upon influenza SOD and CAGR (Figure 4). Cluster 1 included 5 western countries (Belgium, France, Netherlands, Finland, and Sweden), with the highest influenza SOD and second-highest CAGR of influenza SOD, for both population sets. The second cluster included three eastern countries (Bulgaria, Hungary, and Slovakia), with the lowest influenza SOD and CAGR for both population categories. Cluster 3 consisted of the remaining countries, except for Slovenia and Ireland, with low influenza SOD but very high SOD growth rate, which are highly dissimilar to the other EU countries (cluster 4). Cluster 3 was the “average” cluster, with the second-lowest influenza SOD and CAGR, formed of 10 eastern and 7 western countries; see Figure 4A,B.

In the first half of the amalgamation schedule, we found six pairs of western countries, three pairs of eastern countries, and four mixed pairs of western–eastern countries. The highest similarity was recorded for Hungary–Slovakia, and the highest dissimilarity was recorded for Belgium and Bulgaria. Of the four clustering variables, influenza SOD and CAGR for the population above 65 years have slightly higher predictive power for countries’ assignments into clusters (Figure 4C).

## 4. Discussion

Our study shows that EU countries display different health patterns and even country-specific patterns in eastern versus western countries. In eastern countries, populations die mostly of circulatory system diseases, and in Western countries, populations die mostly of malignancy. In eastern countries, incidence of circulatory disease is higher, and in Western countries, incidence of malignancy is higher. We demonstrate that disease SOD in EU populations is heterogeneous. Western, more developed EU countries had higher SOD for almost all comorbidities compared to eastern, less developed countries. Still, their growth rate in eastern EU is higher compared to the western EU. This indicates a higher vulnerability of the EU’s western populations to the COVID-19 pandemic. Significant differences between eastern and western European countries have been demonstrated since five decades ago, regarding death rates, prevalence of comorbidities, and life expectancy [31]. After the creation of the European Economic Community in 1957 and its further expansion, culminating with the European Union established in 1992, one would have expected these differences to fade and to lose strength as a result of economic convergence policies. Still, the average life expectancy is higher in western Europe compared to its eastern part (82.1 against 77.3 years, on average, in 2018), although the western population became younger and more fertile due to migration of the labour force from east to west. At the same time, excess mortality due to malignancy can be detected for all age groups above 65 years in western versus eastern populations. The reverse is true for diseases of the circulatory and respiratory systems—based on SOD for each age group for the population above 65 years [24].

Except SARS and influenza, most epidemics of the 21st century have taken place outside the European continent. Nevertheless, specific measures to prevent influenza and the occurrence of severe forms of these diseases in the general population and the specific vulnerable populations as well as treatment-directed measures have been taken at the European level [20,32]. For example, EU health reports indicate that Hungary, Germany, Greece, Iceland, Slovakia, Malta, Poland, and the Netherlands recommended influenza vaccination for the aged population [11].

Moreover, the comorbidities’ SOD growth rate is higher in the east, so eastern countries are “recovering” the difference from the west. Grouping countries in clusters considering comorbidities’ SOD and their CAGRs demonstrates the presence of a distinctly different vulnerability to diseases in eastern versus western EU countries, except for Sweden, Malta, Finland and Greece, which are included in the eastern category.

The values of excess mortality for malignancy range between 0.28% and 1.57% on average across populations in the two parts of EU, indicating that western populations are more vulnerable than eastern populations to malignancy—calculations based on [24]. Although screening policies are better implemented in western Europe, along with social policies aimed at controlling risk factors and with treatments and health expenditures holding higher shares in GDP, it seems that they have not led to the expected mortality by cancer improvements. For example, lung cancer is the leading cause of death in cancers, and tobacco use is the main risk factor. Joossens et al., in a study based on a questionnaire in 30 European countries, showed that lung cancer prevalence decreases in the United Kingdom, Norway, France, and Ireland as a result of tobacco control policies [33]. Also, breast cancer is among the more prevalent cancers in western European countries, such as Belgium, France, Netherlands, United Kingdom, and the Nordic countries [34]. Screening policies have been introduced for over 20 years. Substantial progress in diagnosis and management of breast cancer as well as the result of health policies are noted, but the incidence of breast cancer is higher in western European countries than in eastern European countries [24].

The excess mortality in Eastern EU for circulatory system diseases ranges between 0.92% and 13.78% (the latter for the 65–69 years age group), showing that older populations in eastern EU are more vulnerable than their peers in western Europe to these diseases—calculations based on [24]. The excess mortality values for respiratory system diseases are much smaller, ranging between 0.36% and 0.90% (eastern against western populations above 65 years) [24]. This indicates a slightly higher vulnerability to these diseases of Eastern EU populations.

Better control of risk factors for cardiovascular disease (sex, age, hypercholesterolemia, cardiovascular disease, high blood pressure, diabetes, and obesity) as well as the response to health policies, better prophylaxis, and more effective treatments were obtained in western European countries. To this, we add policies for screening of cardiovascular diseases and improvements in lifestyle factors (diet and physical activity) [35].

Across the EU, the population above 65 years has higher and statistically significant SODs for COVID-19 comorbidities that are critical to patients’ fate, i.e., cardiovascular and circulatory diseases. At the same time, the SOD means for malignancy and diabetes as well as influenza are statistically significantly higher in the total population compared to population above 65 years, which might indicate cloudy prospects for the current younger EU population at older ages. The European map of most pandemic-threatened aged rural regions [36] is completed by our results that show that EU populations above 65 years had higher SODs for 11/13 comorbidities in Western countries and 8/13 comorbidities in Eastern countries when compared to the total population. The growth rates of comorbidities’ SOD identify malignancy and nervous system diseases as having statistically significant higher SOD increases in the population above 65 years between 2011 and 2016. On the bright side, though, the SOD for blood and cerebrovascular diseases have decreased at a rapid pace in the population above 65 years compared to the total population, which signals increased potential vulnerability to communicable diseases in the current younger population. Also, the growth rate of influenza SOD is higher in the population over 65 than in the total population, which implies that population aging is a significant risk factor for COVID-19 deaths, we note that 19.45% of EU populations (19.88% in the west and 17.77% in the east) were above 65 years in 2017 [24]. This population, with more chronic diseases, is the main vulnerable category to severe forms of the disease and is prone to amount variation due to differences in size and structure from younger population cohorts reaching old age.

Far less than expected, influenza SOD and its growth rate are not perfect predictors of COVID-19 death distribution across EU. Clustering shows that 5 Western EU countries included in cluster 1 (Belgium, France, Sweden, the Netherlands, and Finland) have high SOD and CAGR. Belgium, France, the Netherlands, and Sweden are among the hardest affected countries by the COVID-19 pandemic in deaths per million inhabitants. Surprisingly, some other most-affected countries, Italy and Spain, are in the “average” cluster 3, which might point towards a possible lack of reaction against COVID-19 spread in the initial stages based on lower previous influenza vulnerability.

## 5. Conclusions

Our study argues that a possible explanation for the higher COVID-10 mortality in western compared to eastern EU countries resides in the higher vulnerability of western EU populations to malignancy in COVID-19 comorbidity that has a higher SOD in these countries. Moreover, the cluster that includes 14 western countries has higher average malignancy SOD compared to eastern countries cluster. Worryingly, Europe contained 9% of the world population in 2018 but had a 25% share of the global cancer burden [24,34]; our study adds here that malignancy SOD is higher for the older population in all EU countries, which raises serious health alarms across the EU and signals that this population is at high risk not only from the malignancy burden per se but also from exposure to communicable diseases. The heat maps of EU countries based on populations’ vulnerability to COVID-19 through comorbidities could serve as a model for earliest screening of vulnerable populations (for example, in western EU countries in people with cancer and in eastern countries screening in the population with cardiovascular diseases). Moreover, clinical surveillance for the rapid detection of infected and symptomatic patients in vulnerable populations could more quickly select the group to which applied timely treatment measures are appropriate. Guidelines written in pandemics are of paramount value, but in emergency situations, the time to adapt them to various comorbidities can be a decisive factor in saving lives. Judicial hospitalization based on screening for the vulnerable and infected could more efficiently allocate hospital beds. There is much to be studied, still, about the relationship between COVID-19 and comorbidities and the implications for increasing mortality in vulnerable population.

Although the geographical, historical, religious, and cultural boundaries between eastern and western Europe are hard to delineate, the frontier between east and west for health care system performances is obvious. EU countries were included in 2020 by the World Bank in the high and upper-middle-income categories [37]. Nevertheless, grouping countries into a western category (with more developed and longer-time EU members) and an eastern-located category (including less developed and later EU members) is more useful, given the different institutional characteristics between their health systems [38]. This involves understanding optimal resource allocation within countries, regions, hospitals, and clinics as well as the factors that influence hospital labour and materials supply (in terms of both quantity and quality). It also means planning adaptive policies to respond to the exposure of sections of the broader labour force to COVID-19 and potential similar disease complications. Additionally, economic and socially challenging problems may be avoided by targeting measures in vulnerable populations associated with reasonable measures in the non-vulnerable population (cleaning hands more often than usual, wearing face masks, avoiding meetings, etc.). To our present knowledge, this is the first systematic and statistically enforced proposal towards grasping EU countries’ vulnerability to comorbidities. Comorbidities’ interconnectedness emphasizes the need for an integrated approach.

There are some limitations to our approach. First, raw data are lacking or of limited quality in some countries. Second, although the use of ICD-10 is a long-standing and widely accepted practice, diseases are complex and often attended by coexisting conditions. Moreover, disease interconnectivity makes data on shares of deathless reliability of specific illnesses.

In conclusion, the heat maps of Europe’s vulnerability to diseases based on mortality indicators constitute the basis for more targeted health policy strategies in a collaborative effort at the EU level. In the contemporary, powerful world of science, understanding countries’ specific comorbidities and disease patterns may be a compelling strategy against pandemic wars and not only.

## Figures and Tables

**Table 1:** Comorbidities and causes of death used in the study.

Comorbidity Identified in Tomlins et al. (2020) [12]	Cause of Death—ICD-10 Code	Cause of Death—ICD-10 Code Included in the Research	Cause of Death in the Study
Hypertension	I10–I15 Hypertensive diseases	Other diseases of the circulatory system (remainder of I00–I99)	Other circulatory
Ischaemic heart disease	I20–I25 Ischaemic heart diseases	Ischaemic heart diseases (I20–I25)	Ischaemic heart
Cardiac failure	I50 Heart failure	Other heart diseases (I30–I51)	Other heart diseases
Arrhythmia	I49 Other cardiac arrhythmias	Other heart diseases (I30–I51)	
Valve disease	I30–51 Other heart diseases	Other heart diseases (I30–I51)	
Cerebrovascular	I60–69 Cerebrovascular diseases	Cerebrovascular diseases (I60–I69)	Cerebrovascular
Asthma	J45–46 Asthma and status asthmaticus	Asthma and status asthmaticus (J45–J46)	Asthma
Chronic obstructive pulmonary diseases	J44 Other chronic obstructive pulmonary disease (COPD)	Other lower respiratory diseases (J40–44_47)	Other lower respiratory
Bronchiectasis	J47. Bronchiectasis	Other lower respiratory diseases (J40–44_47)	
Obstructive sleep apnoea	G47.3. Sleep apnoea	Other lower respiratory diseases (J40–44_47)	
Gastrointestinal disease	Disease of the digestive system, unspecified K92.2	Other diseases of the digestive system (remainder of K00–K93)	Other digestive diseases
Endocrine disease	E34–35 Endocrine disorders	Another endocrine, nutritional and metabolic diseases (remainder of E00–E90)	Other endocrine diseases
Obesity	Obesity and other hyperalimentation (E65–E68)	Another endocrine, nutritional and metabolic diseases (remainder of E00–E90)	
Diabetes	Diabetes mellitus (E10–14)	Diabetes mellitus (E10–14)	Diabetes
Malignancy	Malignant neoplasms (C00–C97)	Malignant neoplasms (C00–C97)	Malignancy
Neurological disease	Diseases of the nervous system and the sense organs (G00–H95)	Diseases of the nervous system and the sense organs (G00–H95)	Nervous system
Renal disease	Diseases of kidney and ureter N00–N29	Diseases of the genitourinary system (N00–N99)	Genitourinary
Immunocompromised	D80-D89 Certain disorders involving the immune mechanism	Diseases of the blood and blood-forming organs and certain disorders involving the immune mechanism (D50–89)	Blood
Influenza	J09–J11 Influenza (including swine flu)	Influenza (including swine flu) (J09–J11)	Influenza

**Table 2:** Descriptive statistics of COVID-19 comorbidities shares of death for 28 countries in the European Union—an average of 2011–2016 (percentages).

Diseases	Total Population							Population Aged 65						
	Mean	Median	Minimum	Maximum	SE of Mean	IQR	Std.Dev.	Mean	Median	Minimum	Maximum	SE of Mean	IQR	Std.Dev.
Malignancy	**25.391**	25.491	16.424	31.410	0.643	3.176	3.400	**22.983**	23.105	13.439	28.534	0.650	3.384	3.441
Blood diseases	0.263	0.228	0.054	0.705	0.031	0.240	0.165	0.264	0.219	0.043	0.710	0.033	0.265	0.176
Diabetes	2.246	1.974	0.741	6.595	0.236	1.247	1.247	2.411	2.128	0.749	7.402	0.265	1.388	1.405
Other endocrine	0.553	0.462	0.037	1.506	0.080	0.659	0.425	0.512	0.387	0.027	1.600	0.084	0.652	0.443
Nervous system	3.530	3.125	0.975	14.281	0.491	2.835	2.600	3.692	3.198	0.887	16.486	0.576	3.202	3.049
Ischaemic heart	15.863	13.266	5.987	36.983	1.505	12.545	7.962	17.311	13.792	6.363	44.269	1.831	14.591	9.686
Other heart diseases	8.354	7.638	2.289	22.696	0.818	5.438	4.326	9.114	8.323	1.473	24.385	0.942	6.503	4.983
Cerebrovascular	**9.556**	8.427	5.673	19.613	0.729	4.416	3.857	**10.876**	9.528	6.394	22.423	0.877	5.062	4.643
Other circulatory	6.770	5.439	2.862	18.131	0.739	3.363	3.911	7.645	5.908	3.080	21.550	0.907	3.959	4.799
Asthma	**0.132**	0.120	0.015	0.320	0.012	0.057	0.064	0.136	0.121	0.014	0.378	0.014	0.052	0.074
Other lower respiratory	**2.980**	2.671	0.933	6.565	0.265	1.806	1.404	**3.266**	2.901	0.966	7.190	0.290	1.989	1.535
Other digestive	**2.338**	2.348	1.630	3.700	0.098	0.721	0.518	**2.396**	2.384	1.527	3.899	0.113	0.834	0.600
Genitourinary diseases	**1.751**	1.658	0.642	3.424	0.134	1.026	0.711	**1.989**	1.960	0.732	3.888	0.155	1.299	0.820
Influenza	0.072	0.059	0.009	0.181	0.009	0.060	0.048	0.065	0.050	0.004	0.178	0.010	0.061	0.052

Notes. (1) Bold figures indicate statistically significant means at 5% (*p*-value < 0.05). (2) Causes of death for all European Union countries were collected from the Eurostat and European Mortality Database, according to the codes of the International Classification of Diseases, 10th Revision (ICD-10). They cover all ages and genders, on a population of 507,070,197 persons (mean of 2011–2016). The mean annual total number of deaths (total population) in the European Union for all causes (except injury, poisoning, and certain other consequences of external causes (S00–T98)) is 5,590,916 people. The mean annual number of deaths (2011–2016) in the population above 65 years was 4,132,253. (3) The *t*-test statistic applied to differences in means for each disease for the total population compared to the population aged ≥ 65 years has shown that SOD means are statistically significantly different for 10 out of the 13 COVID-19 comorbidities and for influenza (*p* < 0.05). The exceptions are blood diseases, nervous system diseases, and asthma (*p* > 0.05). The *t*-test was applied for dependent samples.

**Table 3:** Descriptive statistics of COVID-19 comorbidity SOD CAGRs for 28 countries in the European Union—an average of 2011–2016 (percentages).

Diseases	Total Population							Population Aged 65						
	Mean	Median	Minimum	Maximum	SE of Mean	IQR	Std.Dev.	Mean	Median	Minimum	Maximum	SE of Mean	IQR	Std.Dev.
Malignancy	0.123	0.238	−0.873	1.434	0.123	1.093	0.653	0.406	0.553	−0.845	2.224	0.175	1.415	0.925
Blood diseases	−0.356	0.306	−12.237	7.891	0.867	4.684	4.587	−0.615	0.093	−17.871	8.631	1.190	6.015	6.296
Diabetes	2.101	0.704	−4.231	29.480	1.190	3.338	6.295	1.868	0.602	−4.934	29.287	1.205	3.876	6.375
Other endocrine	3.489	3.091	−8.126	12.368	0.947	7.234	5.014	3.689	3.716	−13.003	14.980	1.156	6.427	6.117
Nervous system	4.564	4.109	−0.958	14.782	0.654	4.533	3.460	5.189	4.437	−0.887	17.092	0.848	5.096	4.487
Ischaemic heart	−2.324	−2.538	−4.490	0.550	0.286	2.746	1.512	−2.044	−2.211	−3.902	0.471	0.234	1.968	1.237
Other heart diseases	0.103	0.483	−9.167	5.511	0.686	3.956	3.628	−0.487	0.133	−17.935	5.391	0.899	3.699	4.759
Cerebrovascular	−2.406	−2.374	−6.396	4.352	0.415	2.751	2.198	−2.890	−2.775	−6.635	4.117	0.427	2.659	2.259
Other circulatory	1.033	0.760	−3.568	11.220	0.562	2.709	2.975	0.717	0.362	−5.603	11.710	0.628	3.110	3.325
Asthma	1.569	0.715	−13.648	27.244	1.542	5.207	8.157	2.940	0.861	−10.800	47.750	2.239	6.046	11.845
Other lower respiratory	0.979	1.140	−4.496	8.991	0.553	3.114	2.925	0.624	0.770	−5.150	8.017	0.582	3.254	3.079
Other digestive	0.240	0.155	−4.299	6.736	0.473	2.769	2.501	0.109	−0.259	−5.104	7.863	0.543	3.172	2.871
Genitourinary diseases	1.582	2.098	−10.691	11.418	0.919	6.527	4.865	1.469	1.962	−12.027	11.895	0.988	6.993	5.226
Influenza	13.136	11.272	−30.435	95.694	4.929	28.678	25.613	22.740	23.493	−26.633	126.523	6.101	33.284	31.702

Notes. (1) Causes of death for all European Union countries were collected from the Eurostat and European Mortality Database, according to the codes of the International Classification of Diseases, 10th Revision (ICD-10). They cover all ages and genders, on a population of 507,070,197 persons (mean of 2011–2016). The mean annual total number of deaths (total population) in the European Union for all causes (except injury, poisoning, and certain other consequences of external causes (S00–T98)) is 5,590,916 people. The mean annual number of deaths (2011–2016) in the population above 65 years was 4,132,253. (2) The *t*-test statistic applied to differences in means for each disease for the total population compared to the population aged ≥ 65 years has shown that CAGR means are statistically significantly different for 8 out of the 13 COVID-19 comorbidities and for influenza (*p* < 0.05). The exceptions are other endocrine diseases, other heart diseases, asthma, other digestive diseases, and genitourinary diseases (*p* > 0.05). The *t*-test was applied for dependent samples.

## Data Availability

Data sets used for analysis in the current study are from the European Mortality Database and Eurostat between 2011 and 2016.

## Abbreviations

SARS-Cov2	Severe Acute Respiratory Syndrome Coronavirus 2
COVID-19	coronavirus disease
EU	the European Union
CDs	communicable diseases
NCDs	noncommunicable diseases
ICD-10	the International Statistical Classification of Diseases and Related Health Problems 10th Revision
ICU	intensive care unit
SOD	share of death
CAGR	annual compound growth of rate
